# Characterization of genome-wide genetic variations between two varieties of tea plant (*Camellia sinensis*) and development of InDel markers for genetic research

**DOI:** 10.1186/s12864-019-6347-0

**Published:** 2019-12-05

**Authors:** Shengrui Liu, Yanlin An, Wei Tong, Xiuju Qin, Lidia Samarina, Rui Guo, Xiaobo Xia, Chaoling Wei

**Affiliations:** 1 State Key Laboratory of Tea Plant Biology and Utilization, Anhui Agricultural University, 130 Changjiang West Road, Hefei, China; 2 Guangxi LuYI Institute of Tea Tree Species, 17 Jinji Road, Guilin, China; 3 Department of Biotechnology, Russian Research Institute of Floriculture and Subtropical Crops, Sochi, Russia

**Keywords:** Molecular markers, Genetic diversity, SNP, InDel, Catechin/caffeine biosynthesis, *Camellia sinensis*

## Abstract

**Background:**

Single nucleotide polymorphisms (SNPs) and insertions/deletions (InDels) are the major genetic variations and are distributed extensively across the whole plant genome. However, few studies of these variations have been conducted in the long-lived perennial tea plant.

**Results:**

In this study, we investigated the genome-wide genetic variations between *Camellia sinensis var. sinensis* ‘Shuchazao’ and *Camellia sinensis var. assamica* ‘Yunkang 10’, identified 7,511,731 SNPs and 255,218 InDels based on their whole genome sequences, and we subsequently analyzed their distinct types and distribution patterns. A total of 48 InDel markers that yielded polymorphic and unambiguous fragments were developed when screening six tea cultivars. These markers were further deployed on 46 tea cultivars for transferability and genetic diversity analysis, exhibiting information with an average 4.02 of the number of alleles (*Na*) and 0.457 of polymorphism information content (PIC). The dendrogram showed that the phylogenetic relationships among these tea cultivars are highly consistent with their genetic backgrounds or original places. Interestingly, we observed that the catechin/caffeine contents between ‘Shuchazao’ and ‘Yunkang 10’ were significantly different, and a large number of SNPs/InDels were identified within catechin/caffeine biosynthesis-related genes.

**Conclusion:**

The identified genome-wide genetic variations and newly-developed InDel markers will provide a valuable resource for tea plant genetic and genomic studies, especially the SNPs/InDels within catechin/caffeine biosynthesis-related genes, which may serve as pivotal candidates for elucidating the molecular mechanism governing catechin/caffeine biosynthesis.

## Background

Tea is the most popular non-alcoholic beverage and possesses numerous crucial properties including attractive aroma, pleasant taste, and helpful and medicinal benefits [1–3]. The tea plant (*Camellia sinensis* (L.) O. Kuntze) is a perennial evergreen woody plant (2n = 2x = 30) belonging to the section *Thea* of the genus *Camellia* in the family Theaceae [4, 5]. Evidence is accumulating that the tea plant was originated from Yunnan Province in southwestern China [4–7]. Currently, cultivated tea plant varieties primarily belong to two groups, *Camellia sinensis var. sinensis* (CSS) and *Camellia sinensis var. assamica* (CSA), are extensively cultivated in tropical and subtropical regions around the world [6, 8]. Generally, CSS is a slower-growing shrub with a relatively higher cold-resistance capacity, while CSA is quick-growing with larger leaves and high sensitivity to cold climate [9]. With the successive release of two draft genome sequences, CSA ‘Yunkang 10’ [10] and CSS ‘Shuchazao’ [9], this plant is rapidly becoming another tractable experimental model for genetics and functional genomics research on tea trees. It is known that self-incompatibility and long-term allogamy contributed considerably to the highly heterogeneous and abundant genetic variation of tea plant [11, 12]. Therefore, it is highly important to characterize genome-wide genetic variation between the two varieties.

Molecular markers, based on DNA polymorphisms, are useful and powerful tools for genetic and breeding research. Numerous molecular markers have been successfully developed and applied in genetic and genomic research in tea plant, such as restriction fragment length polymorphisms (RFLPs), amplified fragment length polymorphisms (AFLPs), random amplification of polymorphic DNAs (RAPDs), cleaved amplified polymorphic sequences (CAPS), inter-simple sequence repeats (ISSRs), and simple sequence repeats (SSRs) [12, 13]. With the rapid development of the high-throughput sequencing approaches, the third-generation single nucleotide polymorphism (SNP) and insertion/deletion (InDel) markers are gradually becoming the most widely used molecular markers, demonstrating a promising future in plant genetic and breeding research.

SNPs are the most abundant genetic variations in most plant species, and the exploitation of SNP markers in single-copy regions is considerably easier than use of the other DNA markers [14–16]. InDel markers have practical value for those laboratories with limited resources, which also showed reliable transferability between distinct populations [14, 17, 18]. Both SNPs and InDels have been extensively applied for breeding programs and genetic studies including pedigree analysis, origin and evolutionary analysis, population structure and diversity analysis, construction of linkage maps, QTL mapping, and marker-assisted selection [14, 19–22]. Several studies have also reported the development and application of SNP/InDel markers in tea plant genetic studies. For instance, 16 expressed sequence tag (EST)-SNP based CAPS markers were developed and applied for tea plant cultivar identification [23]. A set of SNPs from EST databases was identified and verified [24]. Fang et al. (2014) validated 60 EST-SNPs, and constructed genetic relationships among tea cultivars and their specific DNA fingerprinting [25]. Based on specific locus amplified fragment sequencing (SLAF-seq), a total of 6042 SNP markers were validated and a final genetic map containing 6448 markers was constructed [26]. Through restriction site-associated DNA sequencing (RAD-Seq) approach, Yang et al. (2016) identified a vast number of SNPs from 18 cultivated and wild tea accessions, and found that 13 genes containing non-synonymous SNPs exhibited strong selective signals suggesting artificial selective footprints during domestication of these tea accessions [27]. By harnessing the two reference genomes, it is now suitable for identifying genome-wide SNPs/InDels between them to guide rapid and efficient development of markers for high-resolution genetic analysis.

The whole genome sequences of tea trees can provide an elegant platform for identifying abundant genetic variation and developing many genetic markers. The completion of the two reference genome sequences is a notable advance for genetic and genomic studies and a basis for this study. The tea plant whole genome CSA ‘Yunkang 10’ was first reported based on the Illumina next-generation sequencing platform, producing a ~ 3.02 Gb genome assembly containing 37,618 scaffolds with N50 length of 449 Kb [10]. Subsequently, the genome assembly of CSS ‘Shuchazao’ was released by combined Illumina and PacBio sequencing platforms, yielding a ~ 3.14 Gb genome assembly that consists of 36,676 scaffolds with N50 length of 1.39 Mb [9]. In this study, several principal objectives were completed. Genome-wide genetic variation and distribution patterns were investigated. A number of polymorphic and stable InDel markers were developed, providing informative molecular markers for genetic and genomic studies. The catechin and caffeine contents of the two tea cultivars were detected, and SNPs/InDels within catechin/caffeine biosynthesis-related genes were characterized. The identified genome-wide genetic variations and newly developed InDel markers provide valuable resources for tea plant genetic and genomic studies, and the identification of SNPs/InDels within catechin/caffeine biosynthesis-related genes can serve as important candidate loci for functional analysis.

## Results

### Mapping of clean reads to the reference genome ‘Shuchazao’

**Fig. 1:**
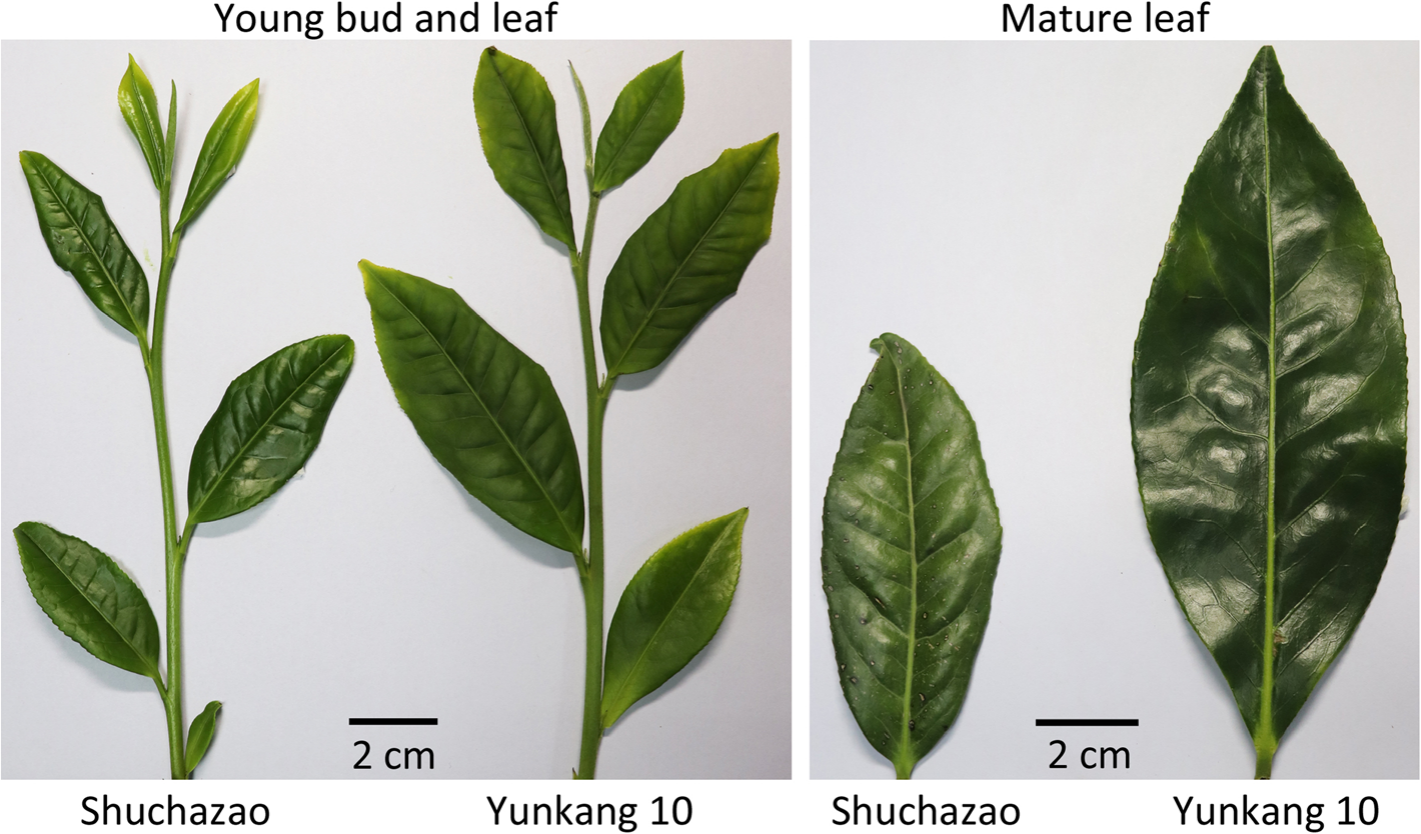
Comparison of bud and leaf size between ‘Shuchazao’ and ‘Yunkang 10’. Young buds and leaves were collected on April 2019, while mature leaves were collected from branches of last-year autumn

CSS ‘Shuchazao’ has been observed to have significant differences in bud, leaf and budding flower size compared with CSA ‘Yunkang 10’ (Fig. 1). The completion of the two reference genome sequences (‘Shuchazao’ and ‘Yunkang 10’) is a notable advance for comparative genomic studies on tea plants in *Thea* section. Therefore, genome-wide genetic variations were identified between the two genome assemblies. After filtering the raw data, a total of 324,154,064 clean reads from the CSA whole genome sequencing data were generated; these reads had a coverage depth of 10.4X the ‘Yunkang 10’ genome with a 100 bp length and 43% GC content. Through alignment, a total of 317,878,025 clean reads were mapped to the reference genome, accounting for 98.1% of total reads. The mapped clean reads contained two types of sequencing reads: pair-end and single-end reads. The former was predominantly type (317,063,284, 99.7%), while single-end reads accounted for only 0.3% (814,741 clean reads).

### Identification and distribution of SNP and InDel loci

**Fig. 2:**
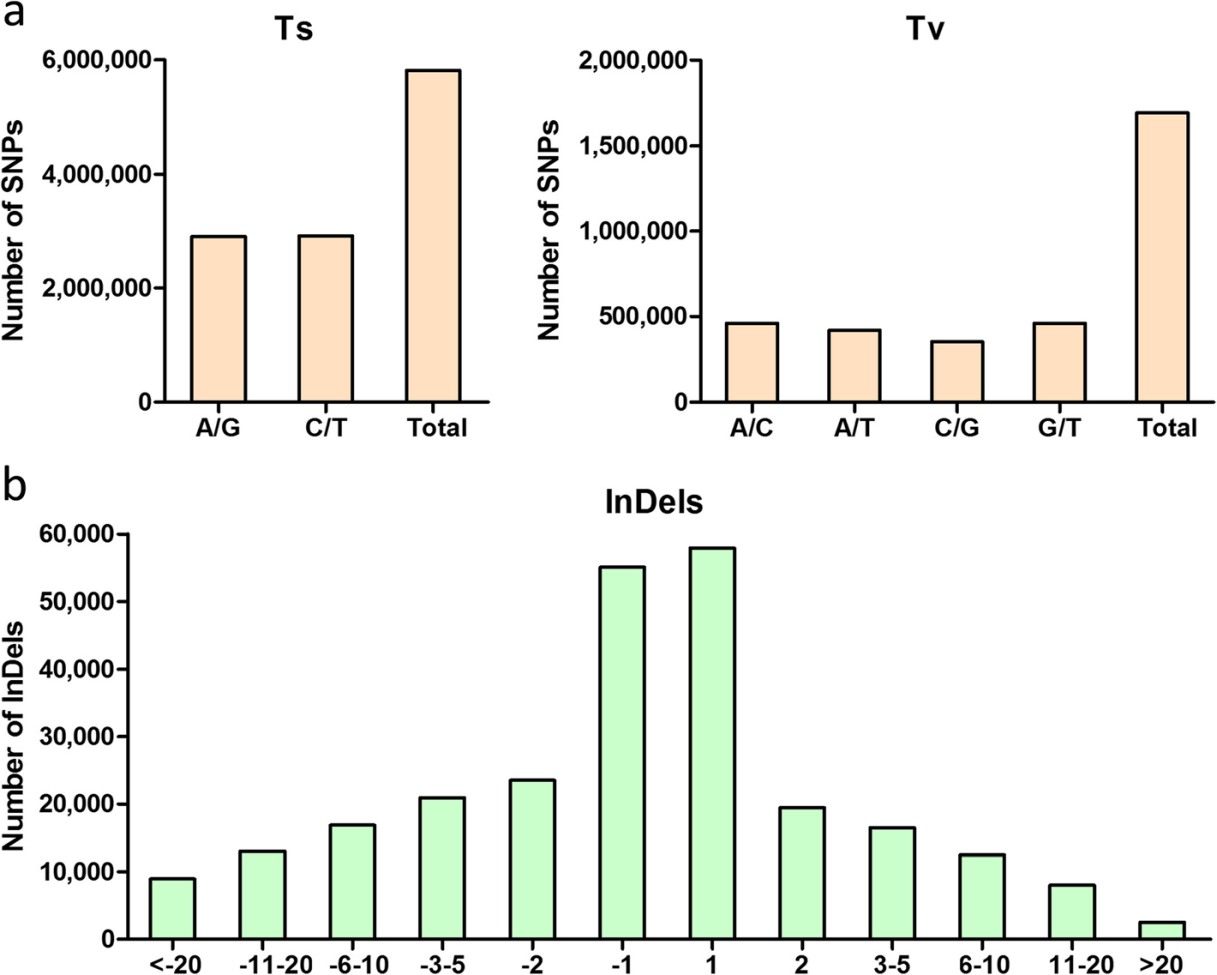
Classification and distribution of identified SNPs/InDels in ‘Yunkang 10’/ ‘Shuchazao’ comparison. **a** Frequency of different substitution types in the identified SNPs; the x-axis and y-axis represent the types and number of SNPs, respectively. **b** Distribution of the length of InDels identified between the two tea cultivars; the x-axis shows the number of nucleotides of InDels, and the y-axis represents the number of InDels at each length

After a series of filtering, a total of 7,071,433 SNP loci were generated, with an average SNP density in the tea genome being estimated to be 2341 SNPs/Mb. Based on nucleotide substitutions, the detected SNPs were classified as transitions (Ts: G/A and C/T) and transversions (Tv: A/C, A/T, C/G, and G/T), which accounted for 77.46% (5,818,773) and 22.54% (1,692,958), respectively (Fig. 2a), with a Ts/Tv ratio of 3.44. In transitions, the number of A/G is equivalent to the C/T type, which included 2,905,203 and 2,913,570, respectively. For transversions, the number of four types (A/C, A/T, C/G and G/T) are almost evenly distributed with an insignificant difference among them, which accounted for 27.23% (460,988), 24.72% (418,536), 20.84% (352,802) and 27.21% (460,632), respectively (Fig. 2a).

A total of 255,218 InDels were identified, with an average density of 84.5 InDels/Mb. The length distribution of InDels was analyzed by dividing the lengths into different groups and calculating the ratios for the corresponding length groups (Fig. 2b). It is obvious that mononucleotide InDels is the most abundant type, accounting for 44.27% (112,976) of the total number. The length of InDels ranging from 1 to 20 bp was predominant, accounting for more than 95.5% (243,749) of the total InDels. A clear tendency was that the number of InDels gradually decreased with increasing InDel length.

### Location and functional annotation of SNPs and InDels

**Fig. 3:**
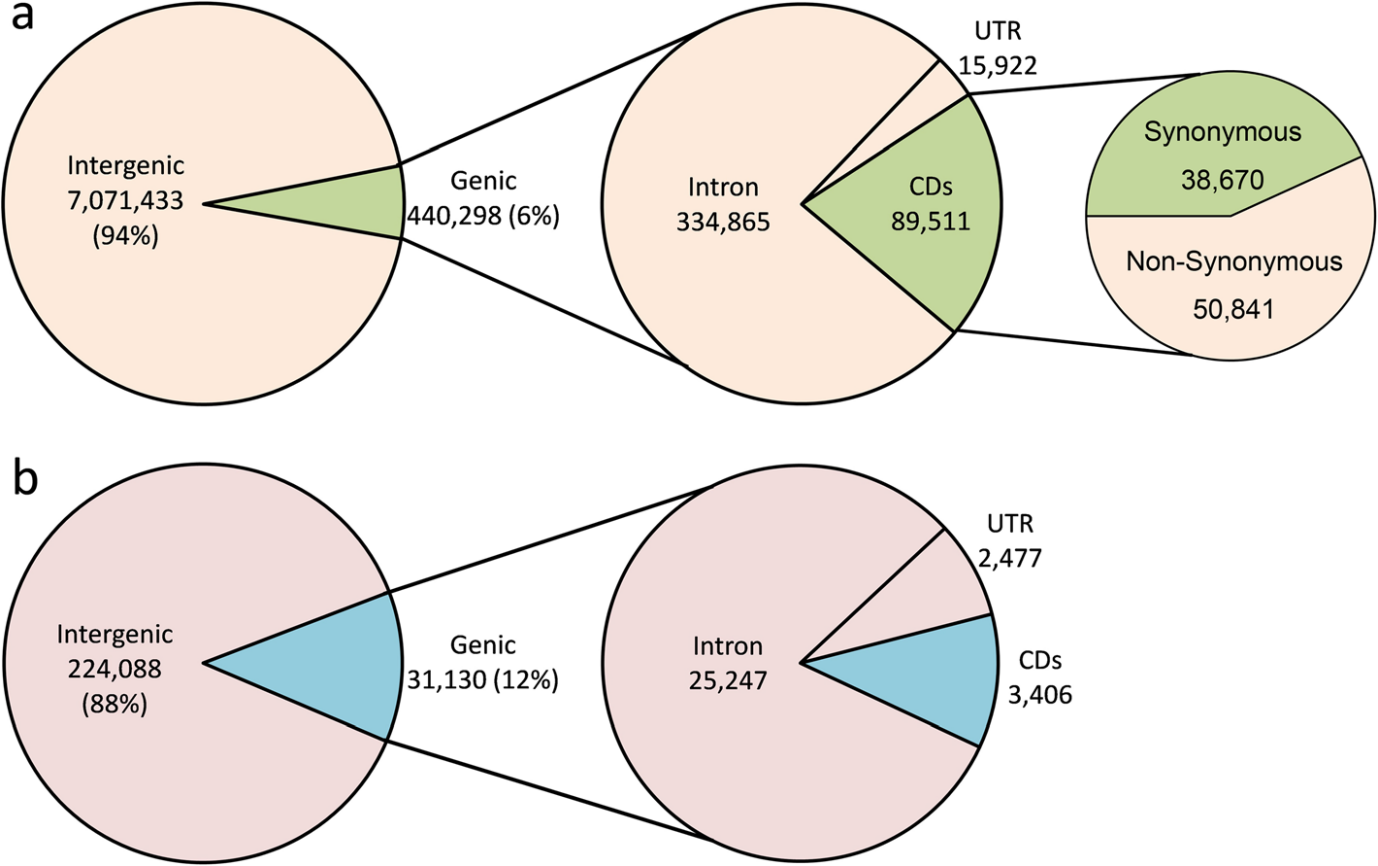
Annotation of SNPs and InDels identified between ‘Shuchazao’ and ‘Yunkang 10’. **a** Annotation of SNPs. **b** Annotation of InDels. SNPs and InDels were classified as intergenic and genic on the ‘Shuchazao’ reference genome, and locations within the gene models were annotated

The annotation of the ‘Shuchazao’ reference genome was used to uncover the distribution of SNPs and InDels within distinct genomic regions. According to the gene structure of the reference genome, the overwhelming number of SNPs (94%) was identified in intergenic regions, while only 6% (440,298) of SNPs were located in genic regions (Fig. 3a). Among the SNPs located in genic regions, 89,511 SNPs were detected in the CDs region, which contained 38,670 synonymous and 50,841 non-synonymous SNPs, respectively. Similarly, a small proportion of InDels were located in the genic regions, which accounted for only 12% (31,130) of the total number (Fig. 3b). Remarkably, 3406 InDels were located in the CDs region, which can be regarded as the preference for developing InDel markers.

To better understand the potential functions of these genetic variations within genes, GO term enrichment analysis of genes containing SNPs/InDels within CDs region was performed. These genes were classified into biological process, cellular component and molecular function categories (Additional file 2: Figure S2). Regarding the genes containing SNPs, the GO terms of cellular process, metabolic process and single-organism process were dominantly abundant in the biological process (Additional file 2: Figure S2A). In the cellular component category, the top three enriched GO terms were membrane, cell and cell part. Based on the molecular function category, catalytic activity and binding are predominantly enriched, while others accounted for a small proportion (Additional file 2: Figure S2A). Interestingly, a nearly consensus result was obtained for GO terms analysis of genes containing InDels, nothing but the number of genes is less compared with the number of genes containing SNPs (Additional file 2: Figure S2B).

### Validation and polymorphism of newly-developed InDel markers

Initially, all InDels were used for designing primer pairs using Primer3.0. To validate the InDels and develop polymorphic InDel markers, we selected 100 InDel markers that were distributed on different scaffolds. To facilitate the screening and development of more practical markers, the lengths of all selected InDels ranged from 5 to 20 bp in length. To determine the reliability and polymorphisms of the primers, six tea cultivars were selected for testing their amplified fragments using Fragment Analyzer™ 96. Of the total primer sets tested, 48 primer pairs were successfully amplified with unambiguous bands and length polymorphisms among the six tea cultivars, 19 primer sets generated non-polymorphic or empty amplifications, and 33 primer pairs yielded non-specific amplification or ambiguous bands. Consequently, the 48 primer sets were regarded as elegant InDel markers and used for further analysis.

**Fig. 4:**
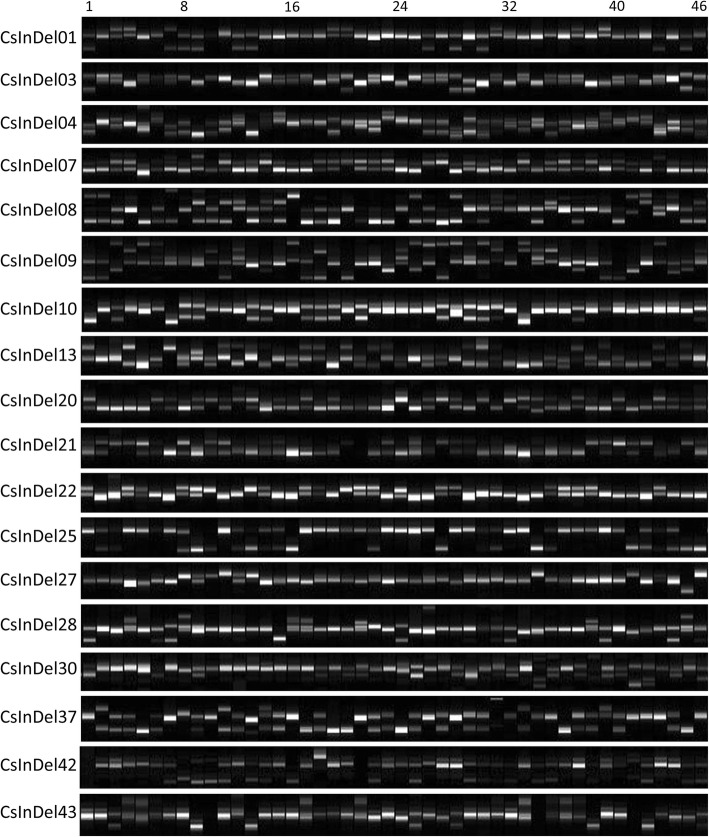
Exhibition of transferability and polymorphism detected by 18 out of 48 InDel markers among 46 tea cultivars

To test cross-cultivars/subspecies transferability, the 48 InDel markers were conducted on a panel of 46 tea cultivars belonging to section *Thea* of genus *Camellia*. The detailed information of the 46 tea cultivars is listed in Additional file 4: Table S1. The results of 18 InDel markers testing on various tea cultivars are shown in Fig. 4, demonstrating that unambiguous and polymorphic bands were obtained based on these markers. The amplified results of the remaining 30 markers were also demonstrated (Additional file 3: Figure S3). For the newly developed markers, 20, 25 and 3 InDel markers generated high polymorphism, moderate polymorphism, and low polymorphism in the 46 tea cultivars, respectively. The PIC value of each InDel marker was presented in Table 1. The amplified allele sizes across them were within the ranges detected in the donor tea cultivar, implying that the amplified fragments were derived from the same loci and that the primer binding sites of the alleles were highly conserved among distinct tea cultivars/subspecies. Several crucial parameters for evaluating polymorphism of markers were subsequently conducted, such as the number of alleles (*Na*) per locus ranged from 2 (CsInDel15, CsInDel16, CsInDel21, CsInDel24, CsInDel25, CsInDel33, CsInDel35, CsInDel39, CsInDel41, CsInDel46, and CsInDel47) to 14 (CsInDel38) with an average of 4.02 alleles, the major allele frequency (MAF) ranged from the lowest 0.266 (CsInDel20) to the highest at 0.957 (CsInDel41 and CsInDel47) with an average of 0.585, the observed heterozygosity (*Ho*) ranged from 0.021 (CsInDel24) to 1.000 (CsInDel15, CsInDel19, and CsInDel29) with an average of 0.524 and the expected heterozygosity (*He*) ranged from 0.082 (CsInDel41 and CsInDel47) to 0.869 with an average of 0.528, the polymorphic information content (PIC) values were from the lowest value 0.078 (CsInDel41 and CsInDel47) to the highest 0.849 (CsInDel38) with an average of 0.457 (Table 1). Notably, the value of *He* has a similar variation trend as the PIC value, while it has a distinct variation trend with *Ho* values. The primer sequences and genomic locations of these newly developed markers are listed in Additional file 5: Table S2. These results showed that these newly developed InDel markers are informative and possess good transferability among various tea subspecies/cultivars.

**Table 1:** Characteristics of 48 newly developed InDel markers

Marker ID	Scaffold location	Fragment size (bp)	*Na*	MAF	*Ho*	*He*	PIC
CsInDel01	Scaffold 5: 236696	139–156	3	0.787	0.383	0.361	0.327
CsInDel02	Scaffold 5: 1208833	186–205	4	0.489	1.000	0.633	0.555
CsInDel03	Scaffold 12: 195263	332–354	3	0.500	0.489	0.577	0.478
CsInDel04	Scaffold 30: 3820588	214–242	5	0.532	0.532	0.636	0.576
CsInDel05	Scaffold 39: 128636	236–264	4	0.479	0.979	0.556	0.448
CsInDel06	Scaffold 41: 2074123	280–295	3	0.808	0.180	0.319	0.273
CsInDel07	Scaffold 46: 249178	176–189	3	0.734	0.362	0.405	0.336
CsInDel08	Scaffold 51: 314982	206–215	6	0.394	0.638	0.691	0.627
CsInDel09	Scaffold 51: 760768	201–248	7	0.532	0.660	0.679	0.645
CsInDel10	Scaffold 52: 469482	288–306	3	0.745	0.255	0.394	0.329
CsInDel11	Scaffold 60: 843530	292–332	6	0.383	0.213	0.748	0.701
CsInDel12	Scaffold 60: 843632	240–275	5	0.426	0.660	0.704	0.645
CsInDel13	Scaffold 64: 151635	270–289	3	0.404	0.617	0.643	0.559
CsInDel14	Scaffold 66: 500052	203–232	4	0.436	0.064	0.621	0.535
CsInDel15	Scaffold 77: 505984	185–207	2	0.500	1.000	0.505	0.375
CsInDel16	Scaffold 89: 1202911	231–248	2	0.819	0.149	0.300	0.252
CsInDel17	Scaffold 98: 664107	306–354	6	0.395	0.256	0.731	0.677
CsInDel18	Scaffold 114: 416691	283–326	6	0.489	0.809	0.703	0.661
CsInDel19	Scaffold 129: 540746	180–214	6	0.422	1.000	0.652	0.579
CsInDel20	Scaffold 154: 767901	285–297	5	0.266	0.979	0.763	0.709
CsInDel21	Scaffold 225: 80286	191–204	2	0.649	0.362	0.461	0.352
CsInDel22	Scaffold 1000: 52494	216–288	3	0.532	0.404	0.612	0.537
CsInDel23	Scaffold 1001: 123324	236–326	6	0.628	0.489	0.568	0.526
CsInDel24	Scaffold 1001: 149678	190–199	2	0.798	0.021	0.326	0.271
CsInDel25	Scaffold 1001: 155681	195–218	2	0.649	0.319	0.461	0.352
CsInDel26	Scaffold 1001: 1251845	341–363	3	0.583	0.833	0.511	0.399
CsInDel27	Scaffold 1001: 1261469	273–290	3	0.777	0.064	0.359	0.306
CsInDel28	Scaffold 1001: 1400899	213–253	6	0.660	0.383	0.537	0.501
CsInDel29	Scaffold 1001: 1491192	182–226	4	0.457	1.000	0.586	0.489
CsInDel30	Scaffold 1001: 1691928	238–258	4	0.745	0.362	0.411	0.363
CsInDel31	Scaffold 1001: 1982826	284–316	4	0.489	0.915	0.619	0.539
CsInDel32	Scaffold 1452: 285463	272–299	3	0.596	0.426	0.511	0.406
CsInDel33	Scaffold 1539: 196438	271–280	2	0.798	0.404	0.326	0.271
CsInDel34	Scaffold 1541: 138532	265–286	3	0.564	0.851	0.523	0.413
CsInDel35	Scaffold 1543: 253456	172–207	2	0.915	0.128	0.157	0.144
CsInDel36	Scaffold 1551: 196819	157–237	3	0.606	0.745	0.499	0.391
CsInDel37	Scaffold 1553: 529121	211–237	4	0.564	0.511	0.547	0.451
CsInDel38	Scaffold 1555: 5209	109–340	14	0.298	0.489	0.869	0.849
CsInDel39	Scaffold 1579: 1466247	261–272	2	0.606	0.787	0.483	0.363
CsInDel40	Scaffold 1592: 672899	276–329	7	0.596	0.979	0.666	0.489
CsInDel41	Scaffold 1593: 1022219	172–187	2	0.957	0.085	0.082	0.078
CsInDel42	Scaffold 1594: 195199	184–206	3	0.691	0.426	0.454	0.380
CsInDel43	Scaffold 1611: 1270988	226–254	5	0.426	0.319	0.684	0.619
CsInDel44	Scaffold 2220: 166816	292–328	3	0.543	0.575	0.521	0.402
CsInDel45	Scaffold 15,285: 211487	281–321	5	0.333	0.952	0.752	0.699
CsInDel46	Scaffold 15,433: 302840	190–253	2	0.638	0.468	0.467	0.355
CsInDel47	Scaffold 15,579: 267174	176–186	2	0.957	0.043	0.082	0.078
CsInDel48	Scaffold 15,650: 137667	228–266	6	0.489	0.596	0.671	0.614
Average	–	–	4.02	0.585	0.524	0.528	0.457

*Na* number of alleles, *MAF* major allele frequency, *Ho* observed heterozygosity, *He* expected heterozygosity, *PIC* polymorphism information content

### Population structure and genetic relationship analysis

**Fig. 5:**
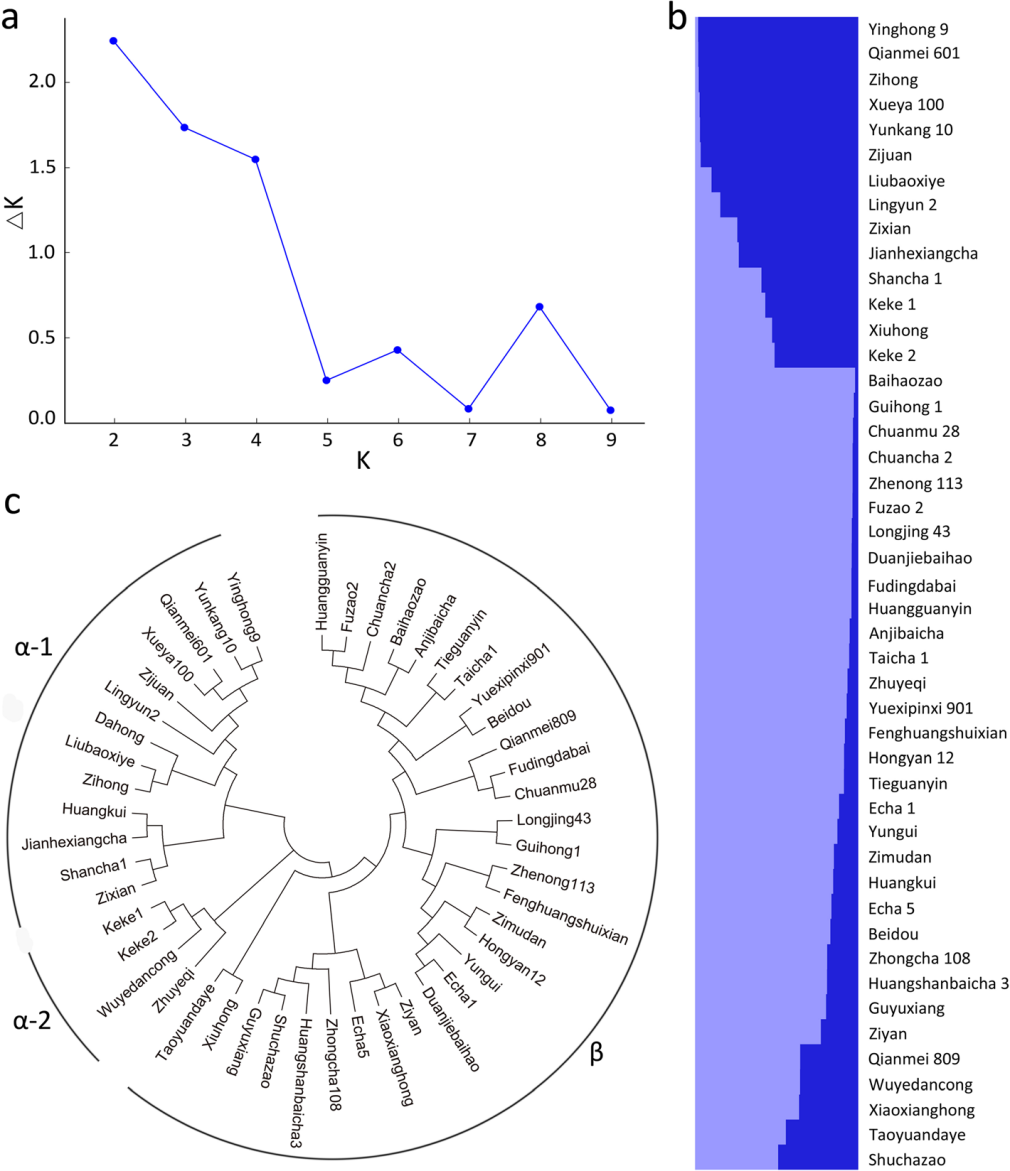
Population structure and phylogenetic relationship analysis based on 48 InDel markers. **a** Estimation of the optimal group number through *ΔK*, the number of *K* was set from 2 to 9. **b** Q-plot of the population structure when *K* = 2. Each tea cultivar is represented by a horizontal bar. **c** The dendrogram was constructed based on genotypes using neighbor-joining algorithm with 1000 bootstrap replicates

Population structure analysis was performed on the 46 tea cultivars using Structure 2.3.3 software based on 48 newly-developed InDel markers. The Q-plot output presented our grouping results, indicating that the two groups were the optimal classification at K = 2 (Fig. 5a). Apparently, tea cultivars from southern and southwestern China (Guangxi, Guangdong, Yunnan and Sichuan Provinces) belonging to *Camellia sinensis var. assamica* were clustered tightly together. In comparison, the tea cultivars possessing smaller leaf sizes and shorter heights that were cultivated in several other provinces were classified into another group (Fig. 5b).

To further confirm the applicability of the developed InDel markers for classification, we constructed a phylogenetic tree based on their genetic distances (Fig. 5c). Two major branches were generated (designated as α and β groups), which contained 17 and 29 tea cultivars, respectively. Group α can be further divided into two subgroups, which were designated as α-1 and α-2 subgroups and consisted of 13 and 4 members, respectively. The dendrogram reflects that the phylogenetic relationships among them are highly consistent with their backgrounds or places of origin, as well as displaying consistency with the results from population structure analysis although a small discrepancy was observed (Fig. 5c).

### Identification of genetic variation in catechin/caffeine biosynthesis-related genes

**Fig. 6:**
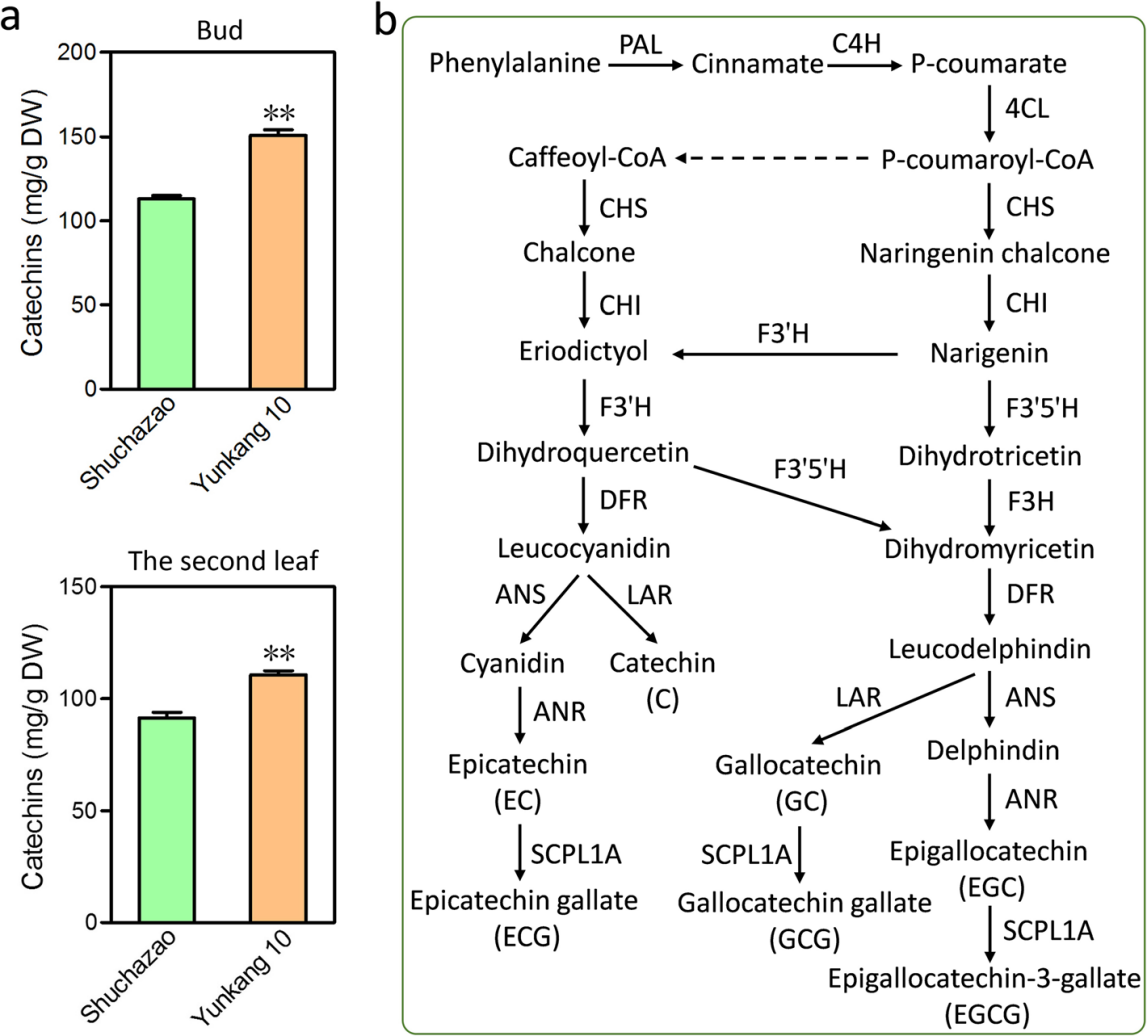
Detection of catechin content and genetic variations within catechin biosynthesis-related genes. **a** Detection of catechin content of the bud and leaf of both ‘Shuchaza’ and ‘Yunkang 10’. T-test was employed for significant analysis and two asterisks represent *p* < 0.01. Each sample was tested with three independent biological replicates and two technical replicates. **b** The flavonoid biosynthesis pathway. PAL, phenylalanine ammonia-lyase; C4H, cinnamic acid 4-hydroxylase; 4CL, 4-coumarate-CoAligase; CHS, chalcone synthase; CHI, chalcone isomerase; F3H, flavanone 3-hydroxylase; F3′H, flavonoid 3′-hydroxylase; F3′,5′H, flavonoid 3′,5′-hydroxylase; FLS, flavonol synthase; DFR, dihydroflavonol 4-reductase; ANS, anthocyanidin synthase; ANR, anthocyanidin reductase; LAR, leucocyanidin reductase; SCLP1A, subclade 1A of serine carboxypeptidase-like acyltransferases

Tea cultivars belonging to *Camellia sinensis var. assamica* possess significant differences in phenotypes (plant height, leaf size and flower) and major characteristic secondary metabolites (such as catechin and caffeine, which contributed tremendously to tea quality) compared with *Camellia sinensis var. sinensis*. Therefore, we detected the contents of catechin (flavan-3-ols) and caffeine in both ‘Shuchazao’ and ‘Yunkang 10’ based on HPLC analysis. The total content of catechin in both buds and the second leaf from ‘Yunkang 10’ was higher than from ‘Shuchazao’ (Fig. 6a). To understand the potential molecular mechanism of difference, we performed the catechin biosynthesis pathway based on several previous studies (Fig. 6b). After search, we identified a number of SNPs and InDels in some crucial genes that are involved in the catechin biosynthesis pathway, including phenylalanine ammonia-lyase (PAL), cinnamic acid 4-hydroxylase (C4H), 4-coumarate-CoA ligase (4CL), chalcone synthase (CHS), chalcone isomerase (CHI), flavanone 3-hydroxylase (F3H), flavonoid 3′-hydroxylase (F3’H), flavonoid 3′,5′-hydroxylase (F3’5’H), dihydroflavonol 4-reductase (DFR), leucoanthocyanidin reductase (LAR), anthocyanidin synthase (ANS), anthocyanidin reductase (ANR), and 1-O-galloyl-β-D-glucose O-galloyltransferase (ECGT, which belongs to subclade 1A of serine carboxypeptidase-like (SCPL) acyltransferases) (Table 2).

**Table 2:** Statistics on SNPs and InDels within catechin biosynthesis-related genes

Gene name	Gene ID	SNP		InDel		Gene name	Gene ID	SNP		InDel	
		DNA	CDs	DNA	CDs			DNA	CDs	DNA	CDs
PAL	TEA014056.1	2	2	0	0	F3H	TEA004906.1	0	0	1	0
	TEA034008.1	6	6	0	0		TEA010326.1	1	1	0	0
	TEA003137.1	16	16	0	0		TEA032907.1	3	3	1	1
	TEA023243.1	3	3	0	0		TEA028622.1	75	1	3	0
	TEA024587.1	3	3	0	0		TEA009737.1	4	4	0	0
	TEA003374.1	2	2	1	0		TEA000753.1	1	1	0	0
C4H	TEA034001.1	16	8	1	1		TEA023937.1	1	1	0	0
	TEA016772.1	5	1	1	0		TEA016601.1	4	2	1	0
	TEA034002.1	6	6	0	0		TEA023790.1	10	3	1	0
4CL	TEA018887.1	1	1	0	0		TEA000474.1	8	1	0	0
	TEA034012.1	9	4	1	1		TEA026443.1	1	1	0	0
	TEA019275.1	14	10	0	0		TEA004898.1	1	1	0	0
	TEA027829.1	12	3	1	0		TEA006643.1	15	15	0	0
	TEA025906.1	2	1	0	0		TEA014951.1	29	8	2	0
	TEA009431.1	42	10	4	2	DFR	TEA032730.1	2	0	1	0
	TEA018045.1	22	3	4	0		TEA023829.1	13	1	0	0
	TEA006577.1	6	1	0	0		TEA021807.1	2	0	0	0
	TEA031627.1	11	8	0	0		TEA021815.1	2	2	0	0
	TEA022274.1	2	1	0	0	ANS	TEA010322.1	1	1	0	0
	TEA010681.1	8	4	0	0		TEA015762.1	1	1	0	0
	TEA002100.1	13	0	1	0		TEA015769.1	1	0	0	0
CHS	TEA018665.1	1	1	0	0	ANR	TEA030023.1	1	1	0	0
	TEA034046.1	34	10	0	0		TEA022960.1	6	2	0	0
	TEA034011.1	6	4	0	0		TEA007646.1	1	0	1	0
	TEA034045.1	1	1	0	0		TEA003247.1	1	1	0	0
	TEA023331.1	2	2	0	0	LAR	TEA021535.1	1	1	0	0
	TEA023340.1	3	3	2	0		TEA027582.1	0	0	2	0
	TEA034013.1	2	2	0	0		TEA009266.1	3	3	1	0
	TEA034043.1	31	7	0	0	SCPLA1	TEA034031.1	4	2	0	0
	TEA034019.1	3	3	0	0		TEA034032.1	11	5	0	0
	TEA034014.1	1	1	0	0		TEA010715.1	6	5	0	0
	TEA011908.1	6	1	0	0		TEA034056.1	33	1	0	0
	TEA019029.1	4	4	0	0		TEA009664.1	4	0	0	0
CHI	TEA034003.1	10	2	1	0		TEA016469.1	2	0	0	0
	TEA033023.1	127	4	10	0		TEA016463.1	9	1	0	0
	TEA033031.1	2	1	0	0		TEA034055.1	59	1	0	0
F3’H	TEA016718.1	2	2	0	0		TEA034034.1	4	0	0	0
	TEA010133.1	5	2	0	0		TEA034036.1	1	1	0	0
	TEA006847.1	14	10	1	1		TEA023444.1	3	0	0	0
F3’5’H	TEA013315.1	12	12	0	0		TEA034039.1	31	2	0	0
	TEA034021.1	6	1	0	0		TEA023451.1	4	1	0	0
	TEA034051.1	32	4	4	0		TEA000223.1	4	0	0	0

**Fig. 7:**
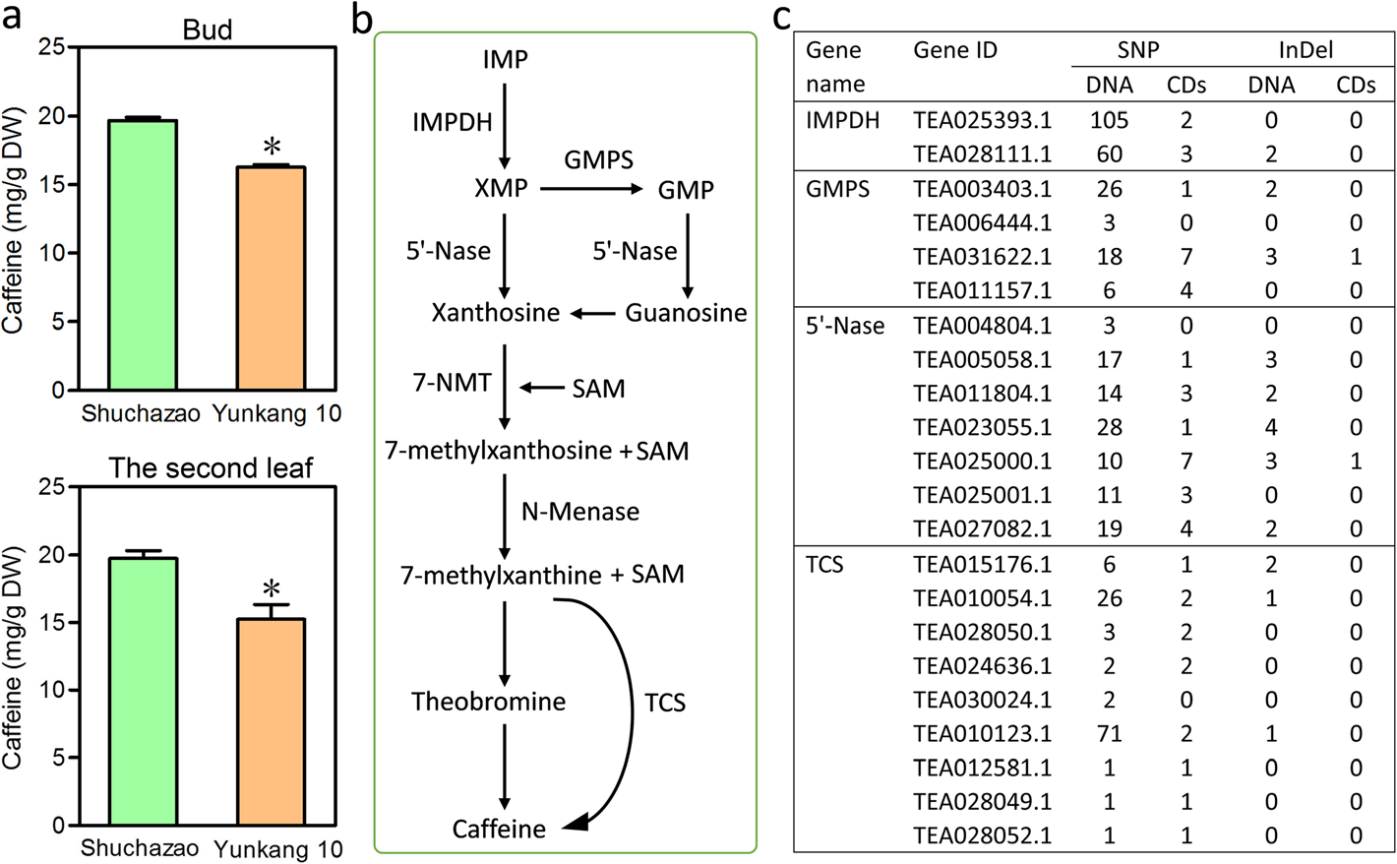
Detection of caffeine content and genetic variations within caffeine biosynthesis-related genes. **a.** Detection of catechin content of the bud and leaf of both ‘Shuchaza’ and ‘Yunkang 10’. T-test was employed for significant analysis and one asterisk represents *p* < 0.05. Each sample was tested with three independent biological replicates and two technical replicates. **b**. The caffeine biosynthesis pathway. IMP, Inosine monophosphate; XMP, Xanthosine monophosphate; GMP, Guanosine monophosphate; IMPDH, IMP dehydrogenase; GMPS, Guanosine synthase; 5′-Nase, 5′-nucleotidase; 7-NMT, 7-methylxanthosine synthase; SAM, S-Adenosyl-L-methionine; N-MeNase, N-methylnucleotidase; TCS, tea caffeine synthase. **c**. SNPs and InDels calling in caffeine biosynthesis-related genes

Detection of caffeine content in the two tea varieties demonstrated that the caffeine in both bud and the second leaf from ‘Yunkang 10’ is lower than that from ‘Shuchazao’ (Fig. 7a). In Fig. 7b, the well-studied caffeine biosynthesis pathway was also performed based on previous studies [10, 28–31]. Similarly, a number of genetic variations within some critical regulatory genes were also detected, such as in IMP dehydrogenase (IMPDH), guanosine synthase (GMPS), 5′-nucleotidase (5′-Nase) and tea caffeine synthase (TCS) genes (Fig. 7c and Table 2). Collectively, these results indicate that certain genetic variations within these genes may explain the significant difference in catechin/caffeine synthesis between ‘Shuchazao’ and ‘Yunkang 10’.

## Discussion

### Identification of genetic variations in tea plant whole genome

The recent release of the ‘Shuchazao’ and ‘Yunkang 10’ genome sequences will strongly facilitate the efficiency of comparative genomics and functional research in tea plants. This advance may enable researchers to study numerous agronomic traits associated with the perennial tea trees with a complete set of tools, including identification and development of SNP/InDel markers. Nevertheless, genome-wide identification and development of SNP/InDel markers are still in infancy, especially genetic variations related to important agronomical traits. By mapping the clean reads of ‘Yunkang 10’ to the reference genome assembly ‘Shuchazao’, we comprehensively surveyed DNA polymorphisms at the genome-wide scale and revealed the high level of genetic diversity between them. The vast number of SNPs and InDels identified in this study will provide valuable resources for tea plant genetics and breeding studies.

After filtering, a total of 7,071,433 SNPs and 255,218 InDels were identified, and their densities distributed in the tea plant genome were estimated to be 2341 SNPs/Mb and 84.5 InDels/Mb, respectively. The densities of SNP and InDel in the tea plant were significant differences compared with in other plant species, such as in *Arabidopsis* [32], *Brassica rapa* [17], quinoa [19], and soybean [33]. These significant differences in SNP/InDel density among different plant species may be due to the distinct filtering protocols and/or the different genomic composition. It is known that tea cultivars belonging to distinct varieties are highly heterogeneous with broad genetic variation due to their self-incompatibility and long-term allogamy [11]. In terms of SNPs, our results showed that A/G and C/T transitions are the most common pattern of nucleotide substitution, which is consistent with the results obtained in other plant species, such as foxtail millet [34], citrus [35], and soybean [33]. For InDels, the most prevalent types in the tea plant genome are short InDels. The number of 1–5 bp InDels is the predominant types, accounting for 76% of all InDels, and similar results were displayed in several other plant species [14, 33–35].

Knowing the genomic positions of genetic variations in genetic markers or functional genes is highly important. It was shown that only minimal SNPs and InDels were distributed in the CDs region, which can be explained by the fact that the CDs region only accounted for a small proportion of the whole genome sequences and had relatively higher conservation compared with other regions. Among the 89,511 SNPs located in the CDs region, a total of 50,841 SNPs were non-synonymous variations. Non-synonymous variations can usually have several functional impacts due to an altered amino acid sequence, such as hampering the interaction between proteins and affecting gene expression due to the functional consequences of distinct motif binding at variation sites [33, 36]. It is worth noting that a total of 3406 identified InDels were located in the CDs region. InDels tend to have more impact on protein structure and function than single base changes, especially those in the CDs region [33]. Nevertheless, genetic variations at UTRs may also play important roles, such as modification of regulatory elements affecting the interaction of the UTRs with proteins and miRNAs [37]. Overall, these SNPs and InDels can serve as important candidates for functional research, especially those InDels in the CDs, which can be considered as a valuable resource for developing phylogenetic and/or functional markers.

### Development and application of InDel markers

Molecular markers are becoming indispensable tools for evolutionary analysis, germplasm identification and conservation, and marker-assisted selection (MAS). SSR is an extensively used marker type among genetic markers, and a large number of highly polymorphic SSR markers have been developed and applied in various genetic studies in tea plants [8, 13]. These SSR markers, however, could easily result in non-specific amplifications and cause confusion in genotyping scoring [19], especially for plant species with large genome and high repetitive sequences. In fact, InDel markers are also PCR-based markers and are similarly affected by genomic complexity. However, they gave relatively less stutter bands due to the variations are more conservative compared with SSR markers [18, 19]. Through a series of screenings, we developed a final of 48 polymorphic and stable InDel markers with 5–20 bp in length based on the genomic assembled sequences (Table 1). The length of fragments of the alleles amplified across tea cultivars was consistent with the expected sizes of the products, implying that the primer binding sites of the alleles were highly conserved. The large proportion of InDel markers displayed a moderate PIC value (0.25 < PIC< 0.5), and the average of PIC was 0.4. It is obvious that the PIC values of most InDel markers were lower than the PIC of the majority SSR markers [2, 8, 38, 39], supporting that the InDel markers are stable and bi-allelic throughout the genome. Therefore, these newly developed InDel markers are suitable for germplasm identification and conservation, genetic diversity analysis, population structure and phylogenetic relationship analysis. In addition, InDels can affect gene functions by causing the gain or loss of a frameshift and/or a stop codon, it is therefore suitable for developing functional markers that might be particularly valuable for MAS [19, 40].

Population structure analysis and phylogenetic trees can reflect the genetic diversity, pedigree relationships, and geographic distances among plant species and/or varieties [2, 16, 22]. They can also be used to evaluate the reliability of molecular markers. To test the reliability and practicability of the newly-developed InDel markers, population structure and phylogenetic relationship analysis were employed, and a consistent result was established (Fig. 5). Apparently, the tea cultivars from southern and southwestern China were clustered together, which originated from *C. sinensis* var. *assamica* populations. In comparison, most tea cultivars from central China had relatively close relationships with each other, which have distinct phenotypes, including small leaf size and short height of tea trees. These results indicate that the population structure analysis and phylogenetic tree reflect the relationships of the 46 tea cultivars, demonstrating the high reliability of these InDel markers for genetic analysis.

### Genetic variations within catechin/caffeine biosynthesis-related genes

Catechin and caffeine are among the most important components in tea plant leaves, which enormously affect the quality of tea products and pharmacy [9, 41]. It is well-known that the contents of catechin and caffeine are influenced by genotypic factors, and significant differences can be observed among distinct tea varieties/cultivars [31, 42, 43].

Based on HPLC detection, we found that the total catechin content from ‘Yunkang 10’ was significantly higher than that from ‘Shuchazao’ in both bud and the second leaf (Fig. 6a). Evidence has shown that the total catechin content of tea varieties tended to decline from the southern to the northern regions [42, 43], and our result is consistent with this tendency. Because catechins are important factors for the oxidation degree and dark tea was produced with severe fermentation during processing [41, 43], our results supported the fact that most tea cultivars belonging to *Camellia sinensis var. assamica* are more suitable for producing dark tea. To understand the potential molecular mechanisms, genetic variations within key genes associated with the catechin biosynthesis pathway were investigated between the two varieties. Unsurprisingly, a large number of SNPs and InDels were identified and some of them were located in the CDs (Table 2). Combining the results of detection of catechin constitutes, it is likely to successfully select certain candidate genetic variations associated with the genotypic factors. For instance, a study reported that a number of candidate allelic variants relating to catechin traits at the F3’5’H locus were identified, and the genetic effects of SNP840/848 were the most robust among them [41].

The result of HPLC detection showed that the caffeine content from ‘Yunkang 10’ was significantly lower than from ‘Shuchazao’ (Fig. 7a). Remarkably, a number of SNPs and InDels were found within some genes associated with the caffeine biosynthesis pathway (Fig. 7c). Previously, a study reported that a 252 bp InDel mutation in the 5′-UTR of TCS1 plays a crucial role in caffeine biosynthesis [44]. Thus, our results can provide valuable candidates for identifying variations within genes related to caffeine biosynthesis. Overall, these valuable resources can be used for further validation, such as functional characterization, association analysis, or development of functional markers for marker-assisted selection.

## Conclusions

Comparison of the whole genome sequences between ‘Yunkang 10’ and ‘Shuchazao’ revealed a large amount of genetic variations, including SNPs and InDels, demonstrating that the tea plant genome is highly variable. The types of SNPs and InDels were subsequently investigated, and their distributions and annotations were also analyzed. Based on these InDel loci, a total of 48 novel InDel markers with moderate polymorphism and high stability were developed. Population structure and phylogenetic relationship analyses were conducted based on these markers, revealing that tea cultivars from *Camellia sinensis var. assamica* were apparently clustered together, while the other tea cultivars from *Camellia sinensis var. sinensis* were clustered into another group. Remarkably, significant differences were observed in catechin and caffeine content between ‘Yunkang 10’ and ‘Shuchazao’, and a number of SNPs and InDels were identified within genes related to the catechin/caffeine biosynthesis pathways.

## Methods

### Plant materials and DNA extraction

A total of 46 clonal tea cultivars were collected from the main tea-growing regions in China, and we obtained permission to collect all the tea samples. The details of these samples, including cultivar name, subspecies, germplasm type, registration number in China and cultivation region are listed in Additional file 4: Table S1. Two individuals (‘Keke 1’ and ‘Keke 2’) were collected from the local natural population in Guangdong Province with the local government’s permission; three clonal tea cultivars (‘Liubaoxiye’, ‘Lingyun 2’ and ‘Zihong’) were collected from the Tea Germplasm Repository of the Tea Research Institute of Guangxi Province with permission; the rest of 41 clonal tea cultivars were commercial cultivars and cultivated widespread in China, which were deposited in the Tea Plant Cultivar and Germplasm Resource Garden in Guohe Town (N31°49′, E117°13′, Hefei, China) of our Institute (Anhui Agricultural University). Until now, a total of 107 national tea cultivars (NTCs) and 139 provincial tea cultivars (PTCs) were registered in China [45]. In this study, 20 NTCs and 13 PTCs were used (the deposition numbers of NTCs are included in Additional file 4: Table S1), and the remaining 13 local tea cultivars (LTCs) were registered by the corresponding provincial government, while the subspecies type of four tea cultivars (‘Keke 1’, ‘Keke 2’, ‘Ziyan’ and ‘Zixian’) was still undetermined.

Young leaves of these tea cultivars were collected and immediately frozen in liquid nitrogen, and subsequently stored at − 80 °C until further use. Total genomic DNA was extracted using the EZgene™ CP Plant Miniprep Kit (Biomiga, USA) following the manufacturer’s protocol. The quality and quantity of DNA samples were determined by 1% agarose gel electrophoresis and the NanoDrop 2000 UV-Vis spectrophotometer, respectively. The concentration of each sample was adjusted to approximately 30 ng/ul for further use in the subsequent PCR amplifications.

### Identification of SNPs and InDels by genome-wide comparison

Considering the quality of genome assemblies of ‘Shuchazao’ is better than the assemblies of ‘Yunkang 10’ [9, 10], it is reasonable to choose the assemblies of ‘Shuchazao’ as the reference genome. The clean reads of ‘YunKang 10’ were retrieved from the NCBI Sequence Read Archive under project number PRJNA381277 (Only the reads with library insert size equal to 500 bp (~ 10 ×) were applied for the further variation calling).

Subsequently, several steps were applied to identify genetic variations between the two assemblies: aligning the clean reads of ‘Yunkang 10’ to the reference using BWA-MEM (version 0.7.17) with parameter ‘-M –R -t 40’, removing PCR duplicates with Picard program, calling SNPs and InDels using GATK-HaplotypeCaller method with parameter ‘-stand_call_conf 30’, and the combination method of Samtools-mpileup with parameters ‘-ugf -t DP -t SP’ and Bcftools-call with parametes ‘-v –m -O’, respectively. Then take the intersection of the two results and use the GATK software to filter according to the following parameters: ‘QD <20.0|| ReadPosRankSum <-8.0|| FS >10.0||QUAL <$MEANQUAL (the first filter)’ and ‘DP < 50.0||GQ <10.0||QD <20.0||FS >200.0||SOR >10.0||MQRankSum <-12.5||ReadPosRankSum <-8.0||QUAL <$MEANQUAL’, finally get a high quality variation locus set (Additional file 1: Figure S1). Annotation for the remaining variations was conducted using snpEFF, and statistics of variations with Vcftools. The genes containing SNPs/InDels in CDs were selected by SnpSift, and their GO term enrichment analysis were performed using the free online platform OmicShare tools (http://www.omicshare.com/tools) (Additional file 1: Figure S1). These software programs have been accurately and expediently applied in SNP calling from next-generation sequencing data [46, 47].

### Validation and development of InDel markers

To develop suitable InDel markers for genetic research, the InDel lengths ≥5 and ≤ 20 bp were used as candidate loci. Specific primers were designed based on the sequences flanking the InDel loci through the Primer 3.0 program with the following parameters: amplicons length (bp) 150–350; primer length 20–22, with the optimum length being 20 bp; *Tm* (°C) 50–60, with 55 °C being the optimum; GC content (%) 40–60, with 50% being the optimum.

A total of 100 primer pairs were randomly selected and preliminarily screened on six tea cultivars (‘Guyuxiang’, ‘Longjing 43’, ‘Echa 5’, ‘Guilv’, Yungui’, and ‘Fudingdabaicha’) using the Fragment Analyzer™ 96 (Advanced Analytical Technologies, Inc., Ames, IA). Primers that gave polymorphic and unambiguous bands were further screened for identification against the 46 tea cultivars. Details refer to PCR reagents and amplification conditions were performed according to our previous study [2]. If more than two fragments were amplified against some individuals using certain markers, only two fragments were collected based on the following criteria: selecting the higher peak value, the higher concentration of amplified products, and the more frequency of fragments occurred among other individuals.

### Genetic diversity analysis

The PROSizeTM 2.0 included in the Fragment Analyzer™ 96 system was applied to visually select strong and clearly polymorphic DNA fragments for scoring, with the same strategy as described previously [8]. The values of expected heterozygosity (*He*) and observed heterozygosity (*Ho*) were determined by Popgene 32 version software. The number of alleles (*Na*), major allele frequency (MAF), and polymorphism information content (PIC) were calculated using PowerMarker 3.25 [48]. Based on the PIC value, markers were divided into three types: highly informative (PIC> 0.5), moderately informative (0.25 < PIC< 0.5) and slightly informative (PIC< 0.25) [19].

### Population structure analysis

Genetic structure analysis of distinct tea accessions was performed using the Structure 2.3.4 program [49]. To minimize Hardy-Weinberg and linkage disequilibrium within each group, the model-based Bayesian clustering algorithm was employed to assign individuals to groups with a predetermined number (*K*, it represents the number of inferred populations). Ten independent runs for each *K* ranging from 2 to 9 were employed and 10,000 iterations were conducted for estimation after a 10,000 iterations burn-in period [19]. Estimation of the subgroups and the best *K* value was performed according to a previous study [50].

### Phylogenetic analysis

Nei’s genetic distances of the 46 tea cultivars based on 48 InDel markers were calculated using PowerMarker 3.25. The dendrogram was constructed using the neighbor-joining (NJ) algorithm as implemented in MEGA 7.0 [51], with bootstrap values at the default setting of 1000 replicates. Pairwise gap deletion mode was employed to guarantee that the divergent domains could contribute to the topology of the tree [52].

### Detection of catechin content using HPLC

The contents of catechin and caffeine were extracted and examined according to the previous study [53]. All samples were detected with three independent biological replicates and each independent sample was examined with two technical replicates. The content of (+)-Gallocatechin (GC), (+)-Gallocatechin gallate (GCG), (−)-Epicatechin (EC), (−)-Epicatechin gallate (ECG), (−)-Epigallocatechin gallate (EGCG), and caffeine were detected. The catechin biosynthesis pathways were established according to previous studies [41, 54–57]. The number of SNP/InDel within the catechin/caffeine biosynthesis-related genes was also identified based on the result of alignment and functional annotation.

## Supplementary information

**Additional file 1: Figure S1.** Flowchart diagram for identifying genome-wide genetic variations between ‘Shuchazao’ and ‘Yunkang 10’ and functional annotation.

**Additional file 2: Figure S2.** Functional categorization of the genes containing genetic variations within the CDs region. **a** Functional annotation of genes containing SNPs within in the CDs region. **b** Functional annotation of genes containing InDels within in the CDs region. These genes were categorized based on GO annotation, and the number of each category is shown based on biological process, cellular component and molecular function.

**Additional file 3: Figure S3.** Exhibition of transferability and polymorphism detected by the remaining 30 InDel markers among 46 tea cultivars.

**Additional file 4: Table S1.** Detailed information for the 46 tea cultivars used in this study.

**Additional file 5: Table S2.** Primer sequences of 48 newly developed InDel markers.

## Data Availability

Most of the important data generated or analyzed during this study are included in the article and its supplementary information files. The other data and materials associated with the current study are available from the corresponding author on reasonable request.

## Abbreviations

AFLPs: Amplified fragment length polymorphisms
CAPS: Cleaved amplified polymorphic sequence
EST: Expressed sequence tag
*He*: Expected heterozygosity
*Ho*: Observed heterozygosity
InDels: Insertions/Deletions
ISSRs: Inter-simple sequence repeats
MAF: Major allele frequency
*Na*: Number of alleles
PIC: Polymorphism information content
RAD-seq: Restriction site-associated DNA sequencing
RAPDs: Random amplification of polymorphic DNAs
RFLPs: Restriction fragment length polymorphisms
SLAF-seq: Specific locus amplified fragment sequencing
SNPs: Single nucleotide polymorphisms
SSRs: Simple sequence repeat
