# Supplementary figures and images for: Characterization of genome-wide genetic variations between two varieties of tea plant (Camellia sinensis) and development of InDel markers for genetic research
Source: BMC Genomics. 2019 Dec 5;20:935. doi: 10.1186/s12864-019-6347-0 (PMC6896268; doi:10.1186/s12864-019-6347-0)

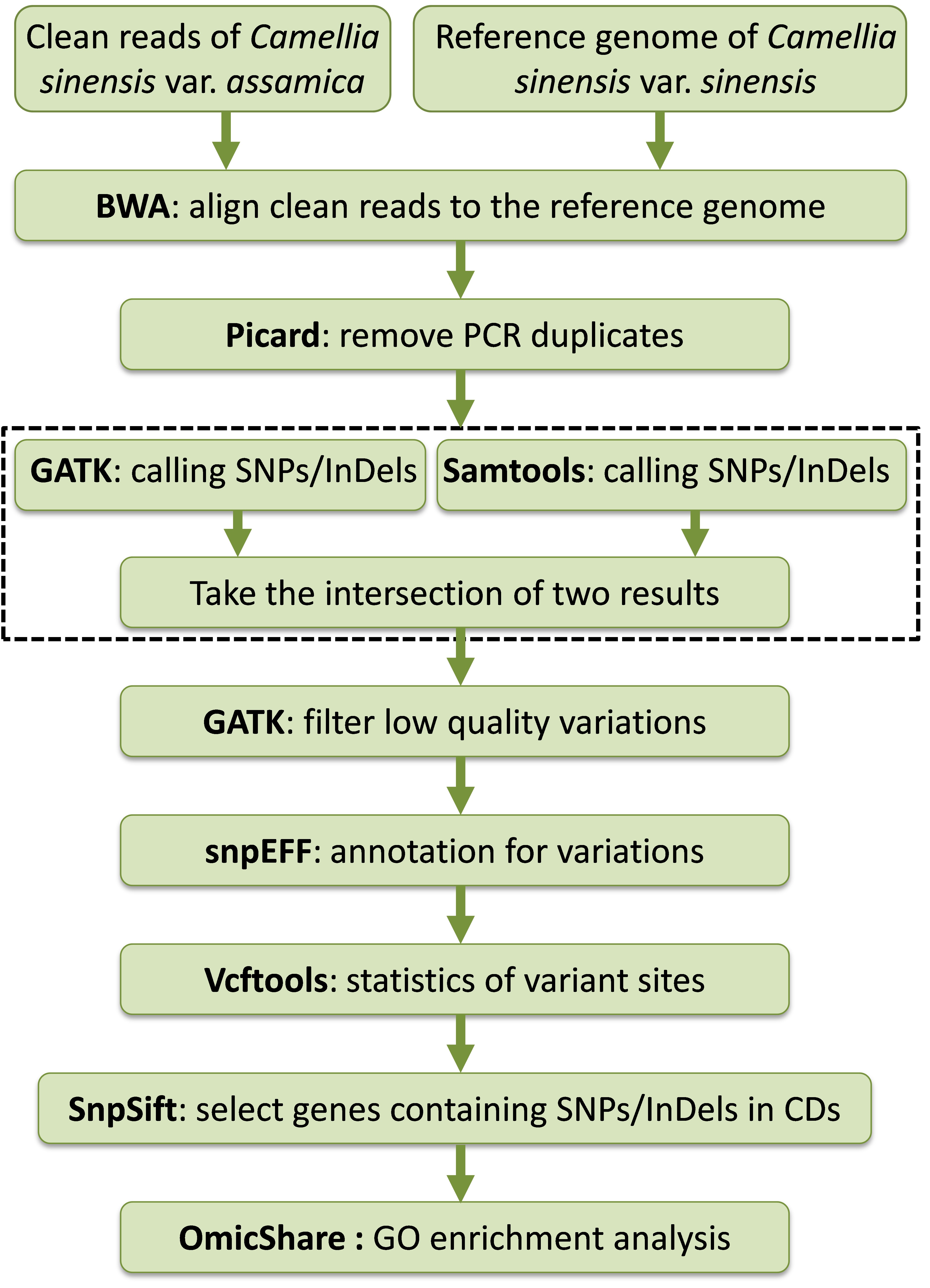

Supplement: Supplementary file 1 — Additional file 1: Figure S1. Flowchart diagram for identifying genome-wide genetic variations between ‘Shuchazao’ and ‘Yunkang 10’ and functional annotation. [file 12864_2019_6347_MOESM1_ESM.jpg]

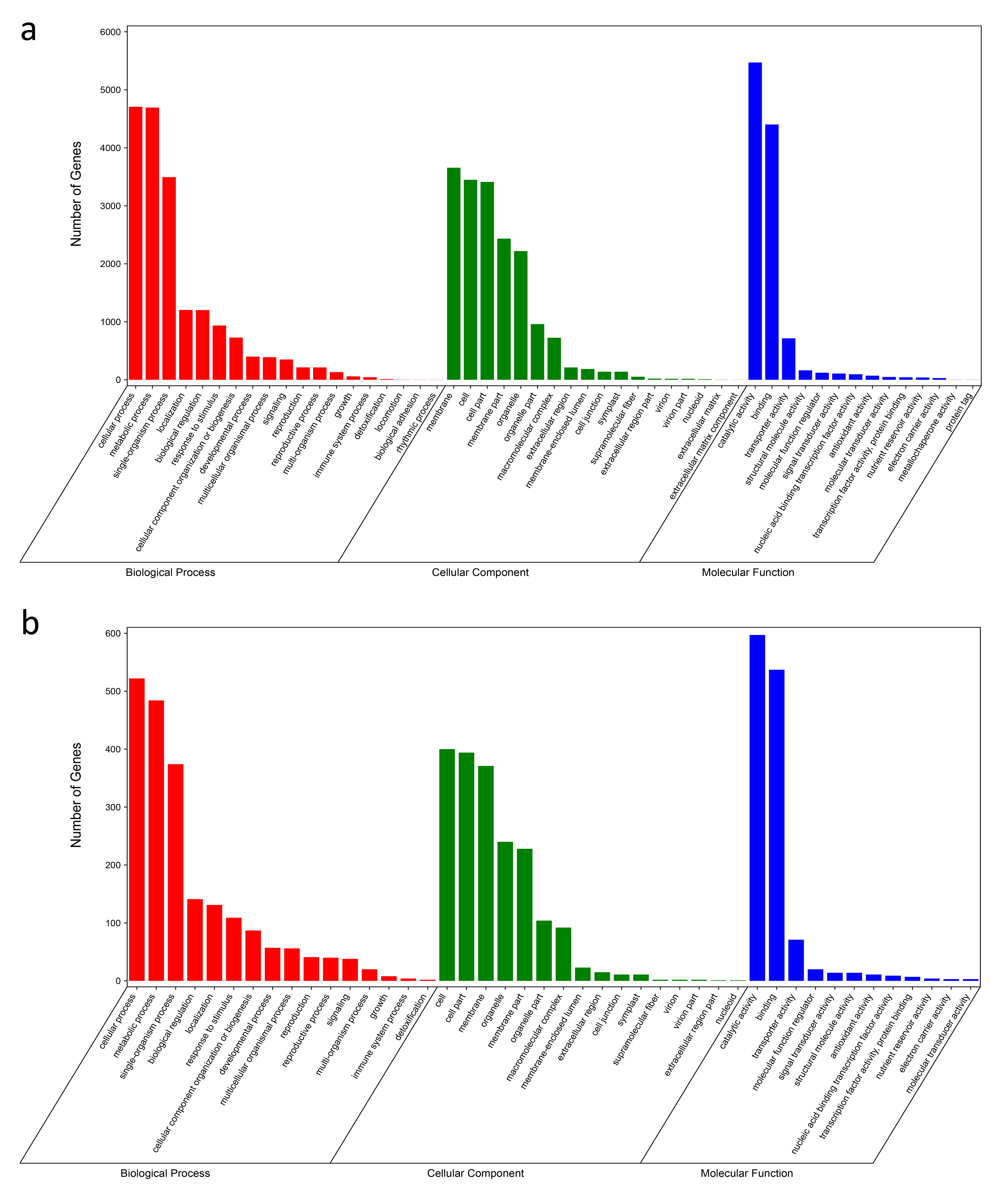

Supplement: Supplementary file 2 — Additional file 2: Figure S2. Functional categorization of the genes containing genetic variations within the CDs region. a Functional annotation of genes containing SNPs within in the CDs region. b Functional annotation of genes containing InDels within in the CDs region. These genes were categorized based on GO annotation, and the number of each category is shown based on biological process, cellular component and molecular function. [file 12864_2019_6347_MOESM2_ESM.jpg]

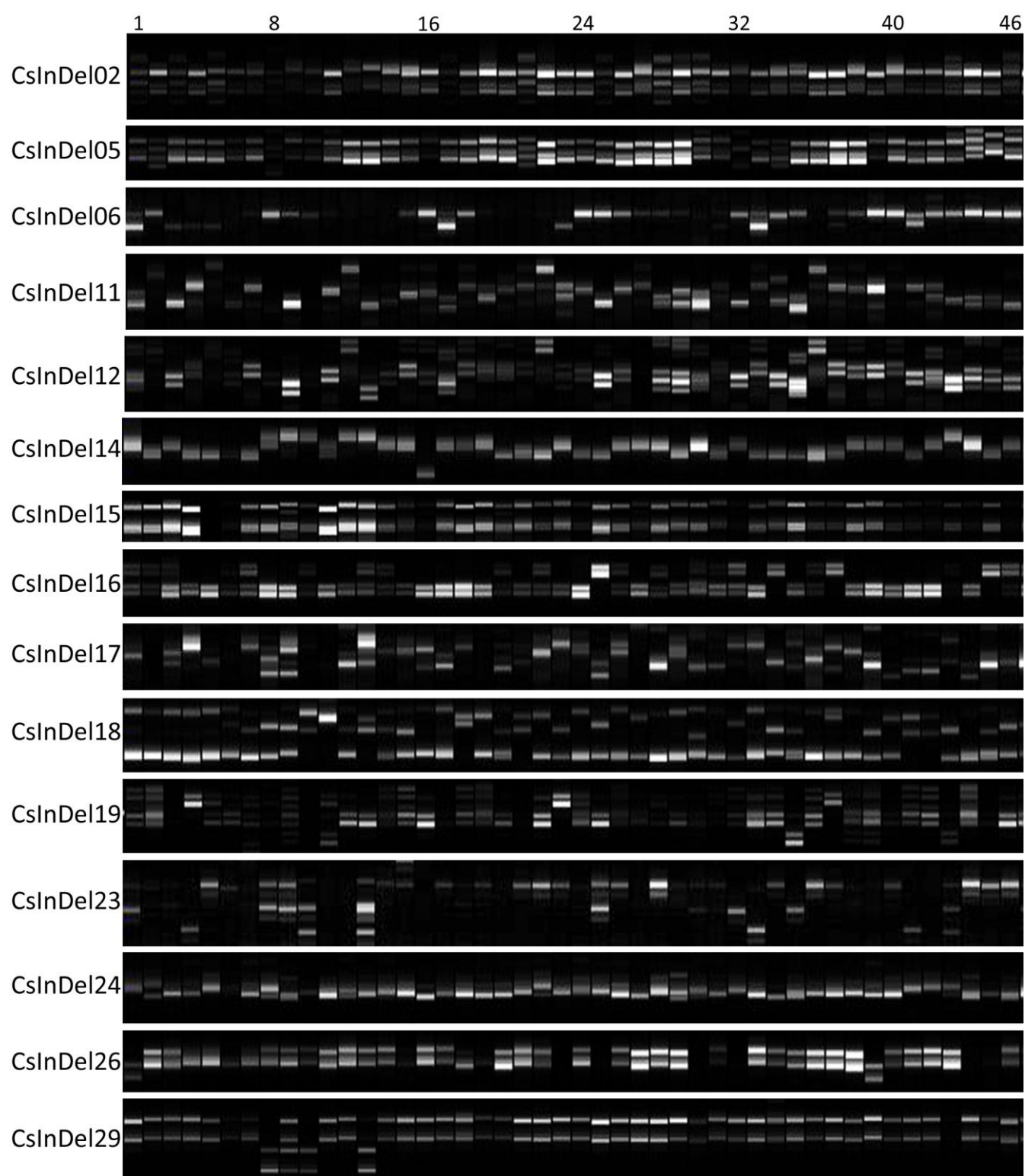

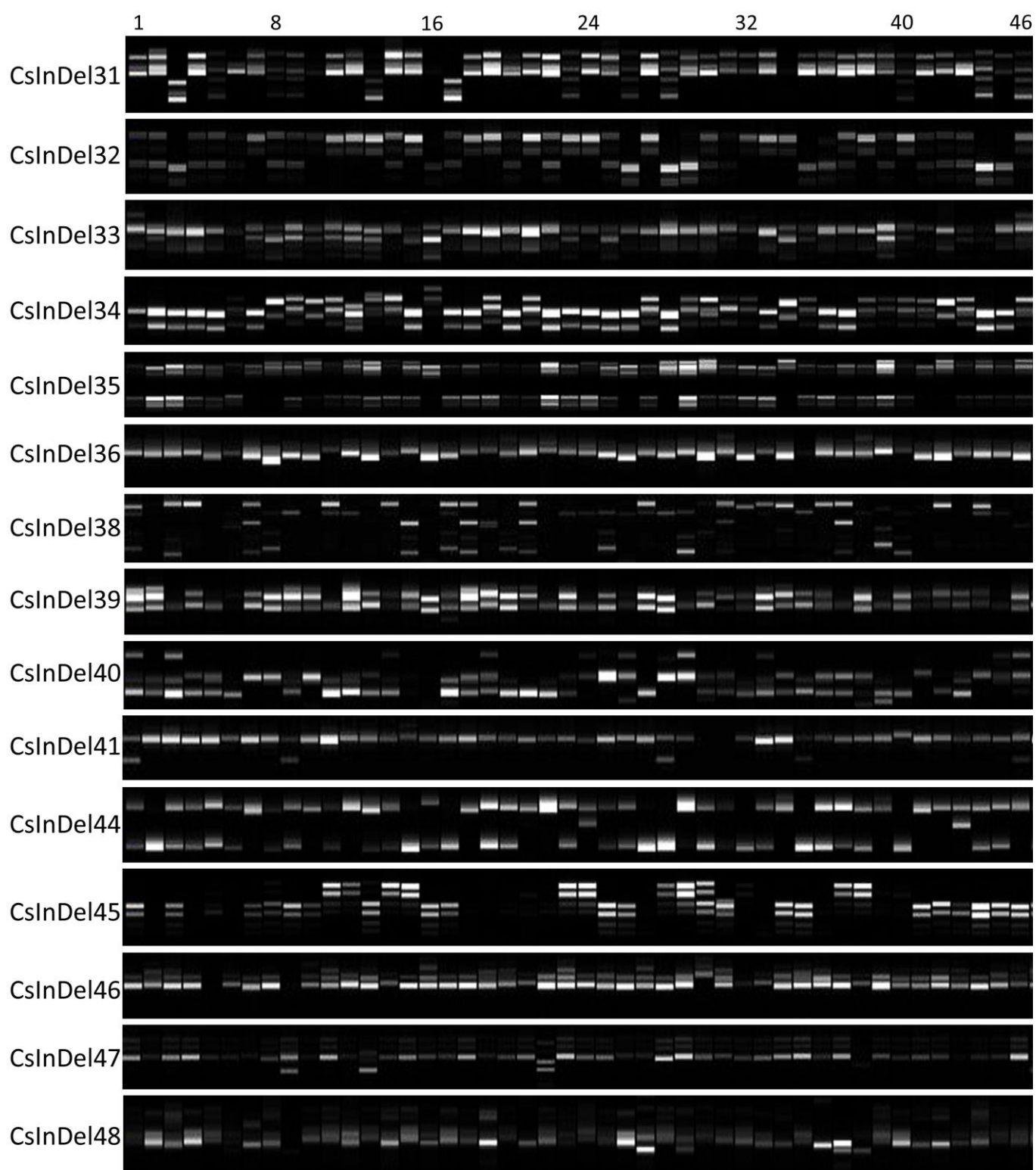

Supplement: Supplementary file 3 — Additional file 3: Figure S3. Exhibition of transferability and polymorphism detected by the remaining 30 InDel markers among 46 tea cultivars. [file 12864_2019_6347_MOESM3_ESM.pdf]
