# Supplementary material for: Characterization of genome-wide genetic variations between two varieties of tea plant (Camellia sinensis) and development of InDel markers for genetic research
Source: BMC Genomics. 2019 Dec 5;20:935. doi: 10.1186/s12864-019-6347-0 (PMC6896268; doi:10.1186/s12864-019-6347-0)
Supplement: Supplementary file 4 — Additional file 4: Table S1. Detailed information for the 46 tea cultivars used in this study. [file 12864_2019_6347_MOESM4_ESM.docx]

**Table S1** Detailed information for the 46 tea cultivars used in this study

| Sample ID | English name | Subspecies | Germplasm type | Registration number in China | Cultivation region |
| --- | --- | --- | --- | --- | --- |
| 1 | ‘Shuchazao’ | *C. sinensis* var. *sinensis* | NTC | GS2002008 | Anhui |
| 2 | ‘Guyuxiang’ | *C. sinensis* var. *sinensis* | LTC | - | Anhui |
| 3 | ‘Huangshanbaicha 3’ | *C. sinensis* var. *sinensis* | LTC | - | Anhui |
| 4 | ‘Baihaozao’ | *C. sinensis* var. *sinensis* | NTC | GS13017-1994 | Hunan |
| 5 | ‘Fuzao 2’ | *C. sinensis* var. *sinensis* | NTC | GS2002001 | Anhui |
| 6 | ‘Huangkui’ | *C. sinensis* var. *sinensis* | LTC | - | Anhui |
| 7 | ‘Qianmei 809’ | *C. sinensis* var. *sinensis* | NTC | GS2002007 | Guizhou |
| 8 | ‘Qianmei 601’ | *C. sinensis* var. *sinensis* | NTC | GS13013-1994 | Guizhou |
| 9 | ‘Yunkang 10’ | *C. sinensis* var. *assamica* | NTC | GS13020-1987 | Yunnan |
| 10 | ‘Xueya 100’ | *C. sinensis* var. *assamica* | PTC | - | Yunnan |
| 11 | ‘Yungui’ | *C. sinensis* var. *assamica* | PTC | - | Yunnan |
| 12 | ‘Xiuhong’ | *C. sinensis* var. *assamica* | NTC | GS2002003 | Guangdong |
| 13 | ‘Yinghong 9’ | *C. sinensis* var. *assamica* | PTC | - | Guangdong |
| 14 | ‘Fenghuangshuixian’ | *C. sinensis* var. *sinensis* | NTC | - | Guangdong |
| 15 | ‘Hongyan 12’ | *C. sinensis* var. *sinensis* | NTC | GS2010020 | Guangdong |
| 16 | ‘Wuyedancong’ | *C. sinensis* var. *sinensis* | PTC | - | Guangdong |
| 17 | ‘Zhongcha 108’ | *C. sinensis* var. *sinensis* | NTC | GS2010013 | Zhejiang |
| 18 | ‘Zhenong 113’ | *C. sinensis* var. *sinensis* | NTC | GS13009-1994 | Zhejiang |
| 19 | ‘Longjing 43’ | *C. sinensis* var. *sinensis* | NTC | GS13007-1987 | Zhejiang |
| 20 | ‘Anjibaicha’ | *C. sinensis* var. *sinensis* | PTC | - | Zhejiang |
| 21 | ‘Fudingdabai’ | *C. sinensis* var. *sinensis* | NTC | GS13001-1985 | Fujian |
| 22 | ‘Huangguanyin’ | *C. sinensis* var. *sinensis* | NTC | GS2002015 | Fujian |
| 23 | ‘Zimudan’ | *C. sinensis* var. *sinensis* | NTC | GS2010026 | Fujian |
| 24 | ‘Tieguanyin’ | *C. sinensis* var. *sinensis* | NTC | GS13007-1985 | Fujian |
| 25 | ‘Jianhexiangcha’ | *C. sinensis* var. *sinensis* | LTC | - | Sichuan |
| 26 | ‘Echa 5’ | *C. sinensis* var. *sinensis* | NTC | GS2010018 | Hubei |
| 27 | ‘Echa 1’ | *C. sinensis* var. *sinensis* | NTC | GS2002013 | Hubei |
| 28 | ‘Shancha1’ | *C. sinensis* var. *sinensis* | PTC | - | Shanxi |
| 29 | ‘Yuexipinxi 901’ | *C. sinensis* var. *sinensis* | LTC | - | Anhui |
| 30 | ‘Taoyuandaye’ | *C. sinensis* var. *pubilimba* | PTC | - | Hunan |
| 31 | ‘Zhuyeqi’ | *C. sinensis* var. *sinensis* | NTC | GS13006-1987 | Hunan |
| 32 | ‘Xiaoxianghong’ | *C. sinensis* var. *sinensis* | PTC | - | Hunan |
| 33 | ‘Guilv 1’ | *C. sinensis* var. *sinensis* | NTC | GS2004001 | Guangxi |
| 34 | ‘Liubaoxiye’ | *C. sinensis* var. *pubilimba* | PTC | - | Guangxi |
| 35 | ‘Lingyun 2’ | *C. sinensis* var. *pubilimba* | NTC | GS13026-1985 | Guangxi |
| 36 | ‘Chuancha 2’ | *C. sinensis* var. *sinensis* | PTC | - | Sichuan |
| 37 | ‘Chuanmu 28’ | *C. sinensis* var. *sinensis* | PTC | - | Sichuan |
| 38 | ‘Taicha 1’ | *C. sinensis* var. *sinensis* | PTC | - | Taiwan |
| 39 | ‘Beidou’ | *C. sinensis* var. *sinensis* | LTC | - | Fujian |
| 40 | ‘Duanjiebaihao’ | *C. sinensis* var. *assamica* | PTC | - | Yunnan |
| 41 | ‘Keke1’ | Unknow | LTC | - | Guangdong |
| 42 | ‘Keke2’ | Unknow | LTC | - | Guangdong |
| 43 | ‘Zijuan’ | *C. sinensis* var. *assamica* | LTC | - | Yunnan |
| 44 | ‘Ziyan’ | Unknow | LTC | - | Sichuan |
| 45 | ‘Zixian’ | Unknow | LTC | - | Sichuan |
| 46 | ‘Zihong’ | *C. sinensis* var. *assamica* | LTC | - | Guangxi |

**Note:** NTC stands for national tea cultivar, registration number [45], PTC represents provincial tea cultivar, and LTC is local tea cultivar.
