# Supplementary material for: Characterization of genome-wide genetic variations between two varieties of tea plant (Camellia sinensis) and development of InDel markers for genetic research
Source: BMC Genomics. 2019 Dec 5;20:935. doi: 10.1186/s12864-019-6347-0 (PMC6896268; doi:10.1186/s12864-019-6347-0)
Supplement: Supplementary file 5 — Additional file 5: Table S2. Primer sequences of 48 newly developed InDel markers. [file 12864_2019_6347_MOESM5_ESM.docx]

**Table S2** Primer sequences of 48 newly developed InDel markers

| Marker ID | Scaffold location | Forward primer | Reverse primer | Region |
| --- | --- | --- | --- | --- |
| CsInDel01 | Scaffold 5: 236696 | ACCACCTCCCCTCATATCACA | ACACTTTAGTCCCTTCCCCCT | CDs |
| CsInDel02 | Scaffold 5: 1208833 | CCCACAGGTTCCACTTCGTAT | ACCTCCATTTAGCAGCTGATCA | CDs |
| CsInDel03 | Scaffold 12: 195263 | TGGAGTCCAACCGTCAATGTT | AGTCCAGGCATCCCAACAATT | CDs |
| CsInDel04 | Scaffold 30: 3820588 | TAATCTGACGCAGCGCTTTTG | GGACCTTTCAGCTTCTCTCCC | CDs |
| CsInDel05 | Scaffold 39: 128636 | CCATCGTCTTCAGCTACAGG | AGGCTGTATCGGGAGGTAAA | CDs |
| CsInDel06 | Scaffold 41: 2074123 | GAGCATACAAACCCACAAGCC | TCTTCGTTTGCTCGTCCCTTT | CDs |
| CsInDel07 | Scaffold 46: 249178 | CATCCACCATCTCCTCCTCT | TTGGTAGTGGTACTGGTGGT | CDs |
| CsInDel08 | Scaffold 51: 314982 | AGACAACTCCGGGTAATGGA | GGCTTGTGGTTCTTCAGGTA | CDs |
| CsInDel09 | Scaffold 51: 760768 | GGTTGTGCAGTTTGGGAGTTG | GGTTGAGGTTGAGGTTGAGGT | CDs |
| CsInDel10 | Scaffold 52: 469482 | GGGGTGTTTGAGATGGGGAAT | GTTGGGATGGTGAGGAGTGAG | CDs |
| CsInDel11 | Scaffold 60: 843530 | GCAGTAGTTGTTGTGGGGAGA | TGGTGGCACTTTGTCTCACTT | CDs |
| CsInDel12 | Scaffold 60: 843632 | TTGATCTTGAAAGGCCTGCC | GGTGGCACTTTGTCTCACTT | CDs |
| CsInDel13 | Scaffold 64: 151635 | GTCCCTCTTCCTCCTCCTTCT | TATCCCCCAACCCAACCTAGT | CDs |
| CsInDel14 | Scaffold 66: 500052 | TATCTCCGAGGCTCCGAAGAT | ACGTCCATGCATTCACACTCT | CDs |
| CsInDel15 | Scaffold 77: 505984 | CGTGTTCTTGAATCCGATGCG | CAATGTCAGCACCCGAAAACC | CDs |
| CsInDel16 | Scaffold 89: 1202911 | TGGAGTGAGGTCGTGAAGAT | AGATCATTACTTGGCACCGC | CDs |
| CsInDel17 | Scaffold 98: 664107 | ATTGAGAATGGCGGAAGTGGT | GGGTTGCAGAGTTAAATTCGGG | CDs |
| CsInDel18 | Scaffold 114: 416191 | GGTTAAGCGTTTGGGTCCTT | ACACACCAACCCTCTCCTTA | CDs |
| CsInDel19 | Scaffold 129: 540746 | CCCTTCGCATATTCCACACA | TGTCATACCCCAGCATCTTG | CDs |
| CsInDel20 | Scaffold 154: 767901 | GTAGTGGGTATGTGCTGGTG | ACCGGGTTTCAAATTGCTTT | CDs |
| CsInDel21 | Scaffold 225: 80286 | ACCATAACAGCAGGCAATCC | AAAGAAACCAGTGGCGGAAT | CDs |
| CsInDel22 | Scaffold 1000: 52494 | GAGGTCGGGGTTGTTACACAT | ATGAGCAATTCCTCCTCGTGG | Intergenic |
| CsInDel23 | Scaffold 1001: 123324 | TCCTACCCTTTCGTTTCCTGC | GAGGGACCGAGCGAAAAGATC | Intergenic |
| CsInDel24 | Scaffold 1001: 149678 | GCACGATTCGCTCTCTTGTTC | AGCGAAATCAACCGTGTACCT | UTR |
| CsInDel25 | Scaffold 1001: 155681 | GGACTTGAGGTGTGCTTTTGG | GGCTTGATCAGAGTCATTCACC | Intron |
| CsInDel26 | Scaffold 1001: 1251845 | GGGACTGTTGGTGTAATTCGC | CATTCGGGCCAAGCTCAAATT | Intergenic |
| CsInDel27 | Scaffold 1001: 1261469 | AGAATGATGTTCGTGTGGCCT | TGGCATTCATAGCGTGTTGTT | Intergenic |
| CsInDel28 | Scaffold 1001: 1400899 | AAAATGAAGAGGAGGGCGACA | AGCCGGCTGTACAAGAAATCA | Intergenic |
| CsInDel29 | Scaffold 1001: 1491192 | ACTCTGCACGGGTATTCATGA | CAAGCCTAGTCATGTGCCTGA | Intergenic |
| CsInDel30 | Scaffold 1001: 1691928 | GGTCAACCTCAACGTCAGCTA | AGGCTTGTTCCCCTTGAGTTT | Intergenic |
| CsInDel31 | Scaffold 1001: 1982826 | AGCAGCTCTACCGGAAATGAA | CCAGGACTACAATCTGCCGAG | Intergenic |
| CsInDel32 | Scaffold 1452: 285463 | TGATCAAACCACCAATGCCA | CGCATGCCATGTTCTTGATT | Intergenic |
| CsInDel33 | Scaffold 1539: 196438 | ACCAACTTGCTCCAAACACA | CCGGAAAGTAGGTCATGGTG | Intergenic |
| CsInDel34 | Scaffold 1541: 138532 | CAGCCGAACTCAACCTATCC | AGCATTTGGAGCCACATTCA | Intergenic |
| CsInDel35 | Scaffold 1543: 253456 | TGAGGCCAAACATGAAGCTT | TCTGATTTTGCTCGTGGTGG | Intergenic |
| CsInDel36 | Scaffold 1551: 196819 | GGATGTTGTAGCTGCCATGA | GCTACCGAATTGCAACCCTA | Intergenic |
| CsInDel37 | Scaffold 1553: 529121 | AAGCAGGCAAATTCAGCAAC | TAACCTTTGATGGCCTGCAA | Intron |
| CsInDel38 | Scaffold 1555: 5209 | AGAGACAGAGAGAAGAGCGA | AAATGGAAAAAGACCGGGGT | Intergenic |
| CsInDel39 | Scaffold 1579: 1466247 | ACGTAACAACCAACCCACTC | TCCATGACACGGGTATCTGA | Intergenic |
| CsInDel40 | Scaffold 1592: 672899 | AGGTACAAACTCCGCTTTTCT | AACTCATTGTACCAGTCCCG | Intergenic |
| CsInDel41 | Scaffold 1593: 1022219 | ATTACTGACTTGATCGCCGG | GGCTCCACTTGCAGAACATA | Intergenic |
| CsInDel42 | Scaffold 1594: 195199 | GTTAAACCTTTGTGCACGGAG | ACTTGAGGTCATTGGCTTGA | UTR |
| CsInDel43 | Scaffold 1611: 1270988 | GCAGGAGACAGATCAGAACG | TGAAAACGGCCATCAAGAGT | Intron |
| CsInDel44 | Scaffold 2220: 166816 | TGTTCTTCTTCTGCTGGGTT | GAACAAGACTGGGGATGTGG | UTR |
| CsInDel45 | Scaffold 15285: 211487 | TCTCTCCAGCCTCATACCAG | CGCTGCAAATAACGACCCTA | Intergenic |
| CsInDel46 | Scaffold 15433: 302840 | GTCTCTTGGGGGAAAACGTT | GTGTTAACCGCCAACCTACA | UTR |
| CsInDel47 | Scaffold 15579: 267174 | CTTGGTGCGGATGAGTTCTT | GTGCAACTCCAAGGAAAAGA | Intergenic |
| CsInDel48 | Scaffold 15650: 137667 | ATCGCACCATAGTCAAACCG | AGTTGGGGTTCACTGGATTG | Intron |
